# Patterns of genetic differentiation in Colorado potato beetle correlate with contemporary, not historic, potato land cover

**DOI:** 10.1111/eva.12757

**Published:** 2019-01-12

**Authors:** Michael S. Crossley, Silvia I. Rondon, Sean D. Schoville

**Affiliations:** ^1^ Department of Entomology University of Wisconsin‐Madison Madison Wisconsin; ^2^ Department of Crop & Soil Sciences, Hermiston Agricultural Research and Extension Center Oregon State University Hermiston Oregon

**Keywords:** agroecosystem, demographic history, genotyping by sequencing, insect pest, land cover, landscape genetics

## Abstract

Changing landscape heterogeneity can influence connectivity and alter genetic variation in local populations, but there can be a lag between ecological change and evolutionary responses. Temporal lag effects might be acute in agroecosystems, where land cover has changed substantially in the last two centuries. Here, we evaluate how patterns of an insect pest’s genetic differentiation are related to past and present agricultural land cover change over a 150‐year period. We quantified change in the amount of potato, *Solanum tuberosum* L., land cover since 1850 using county‐level agricultural census reports, obtained allele frequency data from 7,408 single‐nucleotide polymorphism loci, and compared effects of historic and contemporary landscape connectivity on genetic differentiation of Colorado potato beetle, *Leptinotarsa decemlineata* Say, in two agricultural landscapes in the United States. We found that potato land cover peaked in Wisconsin in the early 1900s, followed by rapid decline and spatial concentration, whereas it increased in amount and extent in the Columbia Basin of Oregon and Washington beginning in the 1960s. In both landscapes, we found small effect sizes of landscape resistance on genetic differentiation, but a 20× to 1,000× larger effect of contemporary relative to historic landscape resistances. Demographic analyses suggest population size trajectories were largely consistent among regions and therefore are not likely to have differentially impacted the observed patterns of population structure in each region. Weak landscape genetic associations might instead be related to the coarse resolution of our historical land cover data. Despite rapid changes in agricultural landscapes over the last two centuries, genetic differentiation among *L. decemlineata* populations appears to reflect ongoing landscape change. The historical landscape genetic framework employed in this study is broadly applicable to other agricultural pests and might reveal general responses of pests to agricultural land‐use change.

## 1 INTRODUCTION

Landscapes can shape genetic variation among the populations of organisms that inhabit them. They can impose natural selection, favoring some alleles while eliminating others, can maintain genetic diversity when they are heterogeneous in space and time, and can promote genetic differentiation by constraining gene flow (Manel & Holderegger, 2013; Schoville, Bonin et al., 2012). However, the effects of landscapes on genetic differentiation can be difficult to quantify due to differences in the spatial and temporal scales at which ecological and evolutionary forces act (Anderson et al., 2010; Epps & Keyghobadi, 2015; Samarasin, Shuter, Wright, & Rodd, 2017). A critical question that is beginning to be assessed by landscape genetic studies is to what extent current patterns of genetic structure reflect legacies of historic landscape structure (Dudaniec et al., 2013; Lee et al., 2013; Oliveira et al., 2018; Storfer et al., 2007; Thomaz, Malabarba, & Knowles, 2017).

Legacies of the effects of historic landscape structure on genetic differentiation might be especially important in agricultural pest systems, where the configuration and composition of agricultural land cover can change rapidly, and the geographic ranges of many insect pests have only recently expanded to encompass agroecosystems (Kirk, Dorn, & Mazzi, 2013). Changes in the structure of agricultural landscapes have been shown to influence genetic diversity in local populations (Crawford, Peterman, Kuhns, & Eggert, 2016; Dixo, Metzger, Morgante, & Zamudio, 2009; Favre‐Bac, Mony, Ernoult, Burel, & Arnaud, 2016) and drive local adaptation to pesticides over short time scales (Crossley, Chen, Groves, & Schoville, 2017; Fritz et al., 2018), but effects on contemporary genetic differentiation among insect populations are limited in taxonomic scope (to bees and grasshoppers; Keller et al., 2013; Jaffé et al., 2016; Suni, 2017) and remain unexamined in insect pest systems. Ignoring the historical landscape context of agricultural pest systems could result in misleading inferences about factors that modulate pest invasions, adaptive evolution, and ultimately give rise to the geographic variation observed in pest traits (Pélissié, Crossley, Cohen, & Schoville, 2018).

Crops have been cultivated for thousands of years in North America (Smith & Yarnell, 2009), but the amount and spatial configuration of cropland began to change substantially in the mid‐1800s (Waisanen & Bliss, 2002). Following European colonization, commercial agriculture moved from East to West, intensifying in the 1900s after improvements in irrigation and fertilizers (Hurt, 2002). Increasing agricultural land cover facilitated range expansions of many insects, and some emerged as serious pests (Kim & Sappington, 2005). One such pest, Colorado potato beetle (*Leptinotarsa decemlineata *Say), a specialist leaf beetle of plants in the family Solanaceae, arose when the staple crop of European American pioneers, the potato (*Solanum tuberosum* L.), reached the Great Plains (Casagrande, 1985; Walsh, 1866). In the United States, the history of *L. decemlineata *range expansion is well documented; the first shift from its ancestral host plant, buffalo bur (*Solanum rostratum *Dunal), to potato was reported in 1859 in central Nebraska (Riley, 1869; Walsh, 1866), and most major potato‐producing regions were subsequently colonized by 1910 (Hsiao, 1985; Tower, 1906). Despite an initially rapid transcontinental invasion, *L. decemlineata *is not a highly dispersive species, being predominantly sessile as larvae, and preferring walking over flight as adults (Boiteau, Alyokhin, & Ferro, 2003; Hare, 1990). A relatively long residence time and low migration rate in agricultural landscapes make *L. decemlineata *a good model to examine how legacies of historical agricultural land cover have shaped contemporary genetic differentiation in insect pest populations. We set out to test whether agricultural production, and how it has changed across time, influences the genetic diversity and genetic structure of the Colorado potato beetle.

One important challenge to detecting landscape effects on genetic differentiation is the potentially confounding effects of populations’ underlying demographic history (Schoville, Lam, & Roderick, 2012). Here, we compared two widely separated landscapes, to examine whether demographic history is a factor influencing observed population genetic structure and variation. We estimated the effects of historical changes in potato land cover on *L. decemlineata *genetic differentiation in the Central Sands of Wisconsin and the Columbia Basin of Oregon and Washington, quantified change in potato land cover at the county level from 1850 to 2012, and compared the relationship between historic and contemporary landscape connectivity and contemporary genetic differentiation among *L. decemlineata *populations. We then used our genetic data to infer changes in *L. decemlineata *effective population size that might have influenced landscape genetic inferences.

## 2 MATERIALS AND METHODS

### 2.1 Beetle sampling

**Figure 1:**
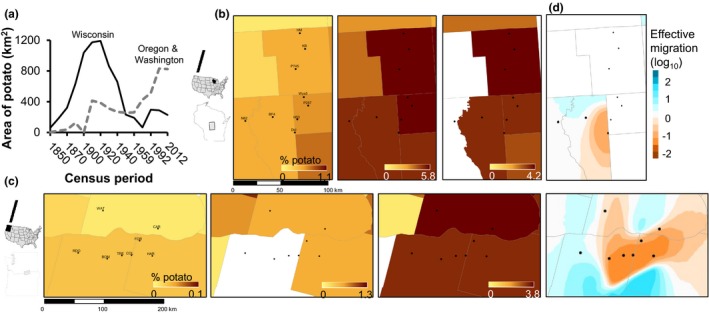
(a) Statewide changes in total potato land cover from 1850 to 2012 in Wisconsin, Oregon, and Washington. (b,c) County maps of potato land cover in 1860, 1910, and 2012 within the study extent in the Central Sands (Wisconsin) and Columbia Basin (Oregon and Washington). (d) Effective migration (posterior mean migration rates on a log_10_ scale) inferred from allele frequency differentiation at 7,408 SNP loci, interpolated over regional study extents

We collected *L. decemlineata* from commercial agricultural fields in eight locations in the Columbia Basin of Oregon and Washington and nine locations in the Central Sands of Wisconsin between 2014 and 2016 (Supporting Information Table S1, Figure 1, Supporting Information Figure S1). These regions differ in landscape composition and the timing of colonization by *L. decemlineata*. The Central Sands is dominated by forest, grassland (or pasture), and corn, while shrubland and wheat are the most abundant land cover types in the Columbia Basin (USDA‐NASS, 2018). However, both landscapes share many less abundant agricultural land cover types in common (e.g., forage and vegetable crops, open water, and developed land cover), providing the opportunity to replicate our analyses. These regions also differ in the timing of *L. decemlineata *colonization: The Central Sands was colonized during the 1860s (Riley, 1869; Walsh, 1866), while the Columbia Basin was colonized after 1910 (Haegele & Wakeland, 1932; Hsiao, 1985). We focused our genetic sampling and analyses of landscape structure and resistance on a 12,855 km^2^ area in the Columbia Basin and an 8,736 km^2^ area in the Central Sands.

Beetle samples from the Central Sands were previously described in Crossley et al. (2017). From each location, we sampled 12 overwintered adult beetles from plants separated by at least three meters. Due to low abundance of *L. decemlineata* in commercial potato fields in the Columbia Basin, four sites represent samples from volunteer potatoes (the result of unharvested tubers from the previous year) in nonhost crops.

### 2.2 DNA sequencing and SNP identification

We isolated DNA from the thoracic muscle tissue of each beetle with the Qiagen DNeasy Blood & Tissue Kit (Qiagen, Valencia, CA) according to the manufacturer's instructions, with the following modifications: We crushed muscle tissue with a sterile micropestle in 180 µl ATL buffer, added 4 µl of RNase A (Qiagen) prior to overnight incubation at 56°C, and eluted DNA from the spin column with one application of 200 µl AE buffer. We then quantified DNA yield and purity with a NanoDrop spectrophotometer (Thermo Fisher Scientific, Wilmington, DE) and by agarose gel electrophoresis. We measured DNA concentrations with a Qubit® fluorometer using the dsDNA High Sensitivity Assay Kit (Life Technologies, Carlsbad, CA) and used 50 ng template for each sample for GBS library preparation. We prepared GBS libraries as described in Elshire et al. (2011); in brief, we digested DNA with the restriction enzyme ApeKI, ligated Illumina adapters and unique barcode adapters (prepared by the University of Wisconsin‐Biotechnology Center) to the digested fragments, and pooled and PCR‐amplified fragments across with the Illumina Solexa PCR protocol. We divided samples among three 96‐well plates, each with a no‐template control, and prepared in three separate GBS libraries (one for the Columbia Basin, two for Central Sands). GBS libraries were sequenced as 150‐bp paired‐end reads, each in one lane of an Illumina HiSeq 2000 (Illumina, San Diego, CA) sequencing system at the University of Wisconsin‐Madison Biotechnology Center. Raw Illumina reads were deposited in the National Center for Biotechnology Information Short Read Archive (accession no. SRP098822 and PRJNA508767).

We demultiplexed paired‐end reads according to unique barcode adapter using the *process_radtags* script from STACKS v1.47 (Catchen et al., 2011). We aligned reads to the *L. decemlineata* reference genome (assembly Ldec_1.5; accession GCA_000500325.1) using BWA‐MEM (Li, 2013), converted SAM files to BAM format with SAMTOOLS (Li et al., 2009), and identified single‐nucleotide polymorphisms (SNPs) with the STACKS pipeline, requiring SNPs to be sequenced to at least 10× depth, and represented in at least half of the individuals per population. We also limited SNP output to one SNP per RAD tag. The total number of SNPs remaining after filtering in *Stacks *was 79,073.

For tests of the effect of landscape resistance on genetic differentiation, we removed SNPs with minor allele frequency less than 5% (reducing SNP set to 10,818) and with greater than 30% missing genotypes among samples (reducing SNP set to 10,403). We then imputed missing genotypes using the haplotype clustering method of BEAGLE 3.3.2 (Browning & Browning, 2007), which replaces missing genotypes with the most frequently observed genotype associated with proximal SNP loci. We then removed SNPs for which over 5% of imputed genotypes had genotype probabilities below 80%, to ensure exclusion of SNPs for which imputation did not add reliable information (Crossley et al., 2017). The resulting dataset consisted of genotypes at 7,408 SNP loci for 93 beetles from the Columbia Basin and 106 beetles from the Central Sands. Because demographic analyses are known to be sensitive to filtering of low‐frequency variants, we also maintained a SNP dataset with no filtering based on minor allele frequency or missing data for demographic analysis. Missing genotypes were imputed using BEAGLE 3.2.2, with no postfiltering. This dataset consisted of 79,073 SNPs, of which, 35,985 were polymorphic among Columbia Basin populations, and 65,535 were polymorphic among Central Sands populations.

### 2.3 Genetic differentiation and diversity

We visualized genetic differentiation over the landscape using estimated effective migration surfaces (EEMS; Petkova, Novembre, & Stephens, 2016), which estimates effective migration rates from genetic distances among population samples, then interpolates values of effective migration over a spatial extent. We created PED files (a standard file format for storing sample genotypes) in R (Supporting Information File S1), converted PED to BED format with PLINK v1.07 (Purcell et al., 2007), and generated genetic distance matrices using the *bed2diff *function of EEMS. We defined the interpolation extents to match the dimensions of the cropped landscape resistance surfaces (described in “Contemporary landscape variables”). We ran EEMS using default MCMC parameters, with 100 demes for each region.

We explored relationships between genetic differentiation, genetic diversity, and potato land cover using linear regressions. We calculated average pairwise *F*_ST_ between sites using the method of Weir and Hill (2002) implemented with *calculate.all.pairwise.Fst() *in the “BEDASSLE” R package (Bradburd, 2014; R Core Team 2017) and calculated nucleotide diversity (π; Nei & Li, 1979) and heterozygosity using the *populations* module of Stacks. We quantified the correlation between genetic diversity and differentiation by regressing average pairwise *F*_ST_ on the average nucleotide diversity between sites and examined relationships between genetic diversity and potato land cover by regressing nucleotide diversity on the proportion county area in potato land cover in 1860, 1910, and 2012 (land cover data described below).

### 2.4 Potato land cover

We tested the association between genetic differentiation among *L. decemlineata* populations and historic and contemporary potato land cover using county‐level agricultural census data (agricultural census records were collated by the United States Department of Agriculture—National Agricultural Statistics Service, Census of Agriculture; tabulated by Haines, Fishback, & Rhode, 2016) that date back to the mid‐1800s. We first visualized spatial and temporal change in potato land cover in the Columbia Basin and Central Sands by generating maps of potato production from 1850 to 2012. For landscape genetic analyses, we represented potato land cover in terms of the proportion of county area (to account for differences in county size), which, in the absence of field‐level land cover configuration data, resulted in conservative estimates of landscape resistance. We obtained county areas from USA county boundary files available from the National Historical Geographic Information System (Minnesota Population Center, 2016) in ArcMap (ESRI) and associated census records of potato production with historic counties using custom R scripts. Because county boundaries changed between 1850 and 2012, we resampled historic potato production maps to match 2012 county boundaries. We did this by calculating the amount of overlap between historic and 2012 county boundaries, then summing the products of the proportion of potato land cover and area of overlap of each section overlapping a 2012 county, according to the following equation:

$$\sum_{i=1}^{n}a_{{}_{i}}\;*\;p_{i}$$

where *n* is the number of counties from the older census period that overlap the 2012 county, *a_i_* is the proportion of county *i *that overlaps the 2012 county, and *p_i_* is the proportion of potato land cover in county *i*. We note that effects of resampling were minimal after the 1910 census, by which time most of the modern county boundaries in our study extent were in place. Potato land cover data are available from Dryad (https://doi.org/10.5061/dryad.93481g3).

Combining information from these maps and historical accounts, we defined three critical transition periods in potato production in the Central Sands and Columbia Basin to include in landscape genetic analysis. First, the census period of 1850 to 1860 captures the steady rise of European American settlement in Wisconsin and the distribution of potato land cover as would have been first encountered by colonizing *L. decemlineata *in the early 1860s (Riley, 1869; Walsh, 1866). Second, the census period of 1900 to 1910 marks the peak in amount and extent of potato production in Wisconsin, while production was just beginning to intensify in Oregon and Washington; 1910 also marks the Columbia Basin landscape as it would have appeared to the first *L. decemlineata *colonists (Haegele & Wakeland, 1932; Hsiao, 1985). Lastly, the census period from 2002 to 2012 depicts the spatial aggregation and local intensification of potato production in contemporary agricultural landscapes; 2012 also captures the steep decline in overall potato production in Wisconsin and its maximum amount and extent in the Columbia Basin.

We modeled landscape resistance to *L. decemlineata *gene flow using a county‐level metric, landscape resistance to transmission (Margosian, Garrett, Hutchinson, & With, 2009), during the three critical periods of 1860, 1910, and 2012. This metric sums the resistance due to the absence of potato (thus assuming potato acts as a conduit to dispersal) along the shortest path between sites, according to the equation,.

$$\text{LRT}\;=\;\frac{1}{\sum_{i\;=\;1}^{n}\left(Z_{i}*\frac{L_{i}}{L_{T}}\right)}$$

where *n* is the number of counties crossed by the shortest path between sites, *Z_i_* is the proportion of county *i *area in potato land cover, *L_i_* is the length (in meters) of the segment of the shortest path line crossing county *i*, and *L*_T_ is the total length of the shortest path line. Values of LRT become large when sites are separated by large spatial extents that lack potato land cover. The assumption that potato acts as a conduit to dispersal is based on the dominance of potato relative to other host plants in agricultural landscapes, and studies documenting up to 75% population reduction when potato fields are rotated as little as 1.5 km away from the previous year's crop (Sexson & Wyman, 2005), suggesting that gene flow is restricted in the absence of potato land cover. Prior to landscape genetic analysis, we standardized resistance distances by dividing by the standard deviation.

### 2.5 Effect of land cover on genetic differentiation

We estimated the effect of landscape resistance on genetic differentiation using the Bayesian Estimation of Differentiation in Alleles by Spatial Structure and Local Ecology framework (BEDASSLE; Bradburd, Ralph, & Coop, 2013), which estimates the effect size of landscape resistance relative to geographic distance on allele frequencies using a Bayesian statistical model. We emphasize, however, that the effects of geographic distance, that is, isolation by distance (Wright, 1943), are not prerequisite to the existence or detection of landscape effects, that is, isolation by environment (Wang & Bradburd, 2014). We tested models estimating landscape effects in each census period separately (1860, 1910, and 2012), and with all census periods included, to account for potential correlations in spatial patterns of potato land cover through time. We ran the beta‐binomial model (*MCMC_BB*) several times initially and adjusted tuning parameters to achieve acceptance rates between 20% and 70%. We then ran 30 independent beta‐binomial Markov chains for four million steps each. We assessed evidence for model convergence by examining trace plots of the posterior probabilities and of the ratios of *αE*/*αD* (effect sizes of landscape resistance and geographic distance) and examining scale reduction factors calculated with a Rubin‐Gelman test (using *gelman.diag()* in the “coda” R package (Plummer, Best, Cowles, & Vines, 2006), which indicates model convergence when the variance in posterior probabilities within Markov chains is equivalent to the variance between Markov chains (upper 95% confidence interval of scale reduction factors approaches one). We assessed statistical significance of differences in effect size ratios among census periods using ANOVA and Tukey's honest significant differences (HSD) at *α* = 5% using the “agricolae” R package (Mendiburu, 2017).

We visualized the relationship between pairwise *F*_ST_ and landscape resistance by first regressing pairwise *F*_ST_ on geographic distance (standardized by dividing by the standard deviation). We then regressed the residuals on landscape resistance (also standardized by dividing by the standard deviation) using linear regression.

### 2.6 Demographic analysis

We used Stairway Plot v2 (Liu & Fu, 2015) to infer the magnitude and timing of changes in effective population size among *L. decemlineata *populations in the Columbia Basin and Central Sands, analyzing each population independently. We generated folded site frequency spectra using minor allele frequencies among the SNPs curated for demographic analysis: 35,985 and 65,535 SNPs among Columbia Basin and Central Sands populations, respectively. We calculated median, 2.5th, and 97.5th percentiles of effective population size among 200 bootstrapped folded site frequency spectra. We defined the sequence length parameter as the number of sites (monomorphic or polymorphic) recovered by *Stacks*; assumed a mutation rate of 2.1 × 10^−9^ per site per generation, based on a recent estimate from the insect *Chironomus riparius *(Oppold & Pfenninger, 2017); and assumed a generation time of 1 year, as is typical of *L. decemlineata *populations in Northern United States (Harcourt, 1971; Voss, Ferro, & Logan, 1988). The magnitude and timing of effective population size changes are sensitive to assumptions about mutation rate and generation time. Unfortunately, there is currently no estimate of genome‐wide SNP mutation rates for the nuclear genome in beetles. Therefore, we employ the molecular clock estimate to compare population trends and the uncertainty around these parameters, rather than infer the size of ancestral populations or any environmental effects on population history. One population from the Columbia Basin exhibited a divergent demographic history and also exhibited the lowest genetic diversity and highest differentiation in pairwise comparisons; thus, we checked the robustness of our landscape genetic associations by repeating analyses with this population excluded.

## 3 RESULTS

### 3.1 Genetic differentiation and diversity

**Figure 2:**
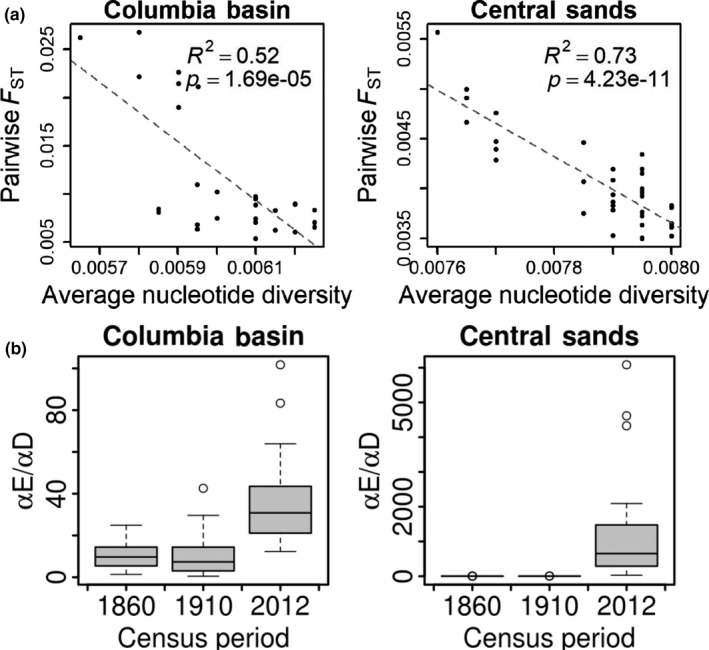
(a) Relationship between genetic differentiation (pairwise *F*_ST_) and diversity (average nucleotide diversity). (b) BEDASSLE estimates of the effect size of landscape resistance (LRT) relative to geographic distance (αE/αD) on allele frequency differences in the Columbia Basin of Oregon and Washington (left) and the Central Sands of Wisconsin (right) in census periods of 1860, 1910, and 2012. Boxplots represent the distribution of final parameter estimates across 30 independent Markov chains run for 4 million steps each

Pairwise *F*_ST_ was generally higher among sites in the Columbia Basin (0.005–0.027) than in the Central Sands (0.004–0.006) (Supporting Information Figure S1). A similar pattern was observed with effective migration (inversely related to *F*_ST_): estimates were generally lower among sites in the Columbia Basin, while most Central Sands sites exhibited high effective migration (Figure 1). Principle components analysis revealed subtle population structuring among sites in both regions, but no complete separation of any sites (Supporting Information Figure S2). Genetic diversity, measured by observed heterozygosity and nucleotide diversity (within each population), was generally lower among populations in the Columbia Basin than in the Central Sands (Supporting Information Figure S1): Average observed heterozygosity across populations was 0.005 (±0.0001) in the Columbia Basin and 0.007 (±0.0001) in the Central Sands, and nucleotide diversity (averaged among populations) was 0.006 (±9 × 10^−5^) in the Columbia Basin and 0.008 (±2 × 10^−5^) in the Central Sands. Pairwise *F*_ST_ was significantly negatively correlated with nucleotide diversity (Figure 2).

### 3.2 Potato land cover change from 1850 to 2012

Potato production began to intensify 50 years earlier in the Central Sands of Wisconsin than in the Columbia Basin of Oregon and Washington, reaching its peak in amount and extent in Wisconsin in 1910 (Figure 1, Supporting Information Figure S3). Both regions exhibited a sharp decline in potato production between 1920 and 1960, then steady increase until 2012. In Wisconsin, potato production became more spatially concentrated in 2012 relative to production levels in 1910, while potato production in Oregon and Washington exceeded that of Wisconsin by 1960 and continued to increase in amount and extent until 2012.

### 3.3 Historic versus contemporary landscape effects

Landscape resistance (LRT) was negatively correlated with geographic distance in the Central Sands, but not in the Columbia Basin, and was highly correlated between years (*R* ^2^ ranging from 52% to 79%; Supporting Information Figure S4). For this reason, we compared models estimating landscape effects in each census period separately with models estimating the effects of all census periods simultaneously. After 4 million steps in 30 independent Markov chains, Gelman‐Rubin tests indicated good model convergence in all but one region‐by‐year comparison (upper 95% confidence intervals of scale reduction factors between 1.2 and 1.3 except the Columbia Basin in 1910 [=1.8]); but the range of parameter estimates for this model was relatively narrow (αD/αE between 0.5 and 42.6). Effect sizes of geographic distance and landscape resistance on allele frequency covariance were generally low, ranging from 10^−4^ to 6 and centered at 10^−3^ for geographic distance and ranging from 10^−3^ to 4 and centering at 10^−2^ for landscape resistance. In both regions, the average relative effect of landscape resistance (LRT) was significantly higher (at *α *= 5% level) in 2012 compared to 1860 and 1910 (Figure 2, Table 1). This result was robust to inclusion of all census periods in the same model (Supporting Information Figure S4, Table 1), indicating that the effects of contemporary potato land cover were distinct from correlated patterns of historical potato land cover. There was no signal of isolation by distance in either region (Supporting Information Figure S5).

**Table 1:** Summary of effect sizes of landscape resistance to transmission of potato land cover in 1860, 1910, and 2012, relative to geographic distance on allele frequency differences among *Leptinotarsa decemlineata* populations in the Columbia Basin (Oregon and Washington) and Central Sands (Wisconsin). Means and standard errors were taken across 30 independent MCMC chains consisting of 40 million steps each. HSD group denotes significantly different αE/αD ratios at *α* = 5%

Region	Year	Mean αE/αD	Standard Error	HSD group
Analysis of each year independently				
Columbia Basin	2012	35.4	3.8	a
	1910	10.9	1.8	b
	1860	10.9	1.2	b
Central Sands	2012	1,155.3	1,440.5	a
	1910	1.3	1.3	b
	1860	1.0	0.8	b
Analysis with all years included				
Columbia Basin	2012	67.9	12.8	a
	1910	8.7	1.6	b
	1860	5.1	1.0	b
Central Sands	2012	80.1	16.0	a
	1910	1.8	0.4	b
	1860	1.4	0.3	b

There was a slight negative correlation between residual pairwise *F*_ST_ (after accounting for effects of geographic distance) and landscape resistance (Supporting Information Figure S5), but this was only statistically significant in the Columbia Basin. The trend in the Columbia Basin was highly sensitive to the inclusion of one site that exhibited higher values of pairwise F_ST _and lower genetic diversity (Supporting Information Figure S5). We found no significant correlations between nucleotide diversity and the proportion county area in potato land cover surrounding each site.

### 3.4 Demographic analysis

**Figure 3:**
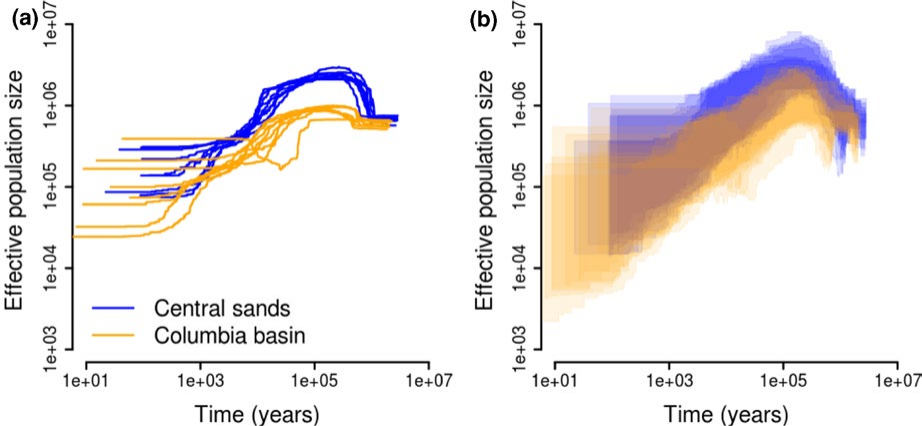
Stairway plots depicting (a) median and (b) 2.5th‐97.5th percentiles of effective population size among 200 bootstrapped folded site frequency spectra. Inferences were based on the folded site (minor allele) frequency spectra for each *Leptinotarsa* *decemlineata *population, length of genomic coverage by genotyping‐by‐sequencing reads, an assumed mutation rate of 2.1 × 10^−9^ per site per generation, and a generation time of one year

Despite the differences observed in population genetic structure and site‐specific genetic diversity, we found similar histories of effective population size change among *L. decemlineata *populations in the Columbia Basin and Central Sands, with some minor population‐specific differences (Figure 3 and Supporting Information Figure S6). The 2.5th–97.5th percentiles of effective population size generally overlapped between Columbia Basin and Central Sands populations, with the largest separation occurring between 20–400 kya, when the highest estimate of median effective population size among Columbia Basin populations was ~1 million, and the lowest in the Central Sands was ~2 million. The most recent estimates of effective population size date from 10 to 100 years ago, and range from 25,000 to 400,000 individuals (Figure 3), though there was high uncertainty around these values. One population from the Columbia Basin had a distinct demographic history, evident from the folded site frequency spectrum (Supporting Information Figure S7; population name “Tree”) and stairway plots. This population experienced a decline in effective population size earlier than other populations, followed by recovery to 60% of its ancestral size.

## 4 DISCUSSION

Potential effects of historic landscapes on contemporary patterns of genetic structure are often acknowledged, but rarely tested explicitly. This could have important implications for improving land management decisions and explaining changes in pest dynamics through time. We leveraged historic agricultural census data to test whether patterns of genetic structure in an agricultural insect pest were more related to past than present land cover configuration in two landscapes.

### 4.1 Historic versus contemporary landscape effects

We observed distinct histories of land cover change in our study areas: Potato land cover increased and achieved its climax earlier in the Central Sands (1910 census period) than in the Columbia Basin (2012). While potato land cover has decreased and become spatially concentrated relative to historic baselines in Wisconsin, it continues to increase and expand in the Columbia Basin. The differences in the history of land cover change in these regions provided an ideal opportunity to detect a legacy of historic land cover on contemporary patterns of genetic differentiation.

Overall, we found weak associations between landscape resistance and genetic differentiation, but when contemporary and historic landscape effects were compared, contemporary land cover had a larger relative effect size in both study regions. This suggests that contemporary landscape structure is weakly limiting effective gene flow among *L. decemlineata *populations. While the effects remain weak, importantly this result suggests that land‐use decisions and landscape management could be useful tools to reduce pest movement, alter patterns of genetic diversity and potentially slow adaptation, especially for Colorado potato beetle, whose dispersal and abundance are sensitive to crop land cover composition (Huseth, Frost, Knuteson, Wyman, & Groves, 2012). Furthermore, our results suggest that legacies of historical potato land cover are relatively less important than contemporary land cover for understanding patterns of genetic differentiation among these *L. decemlineata *populations. Importantly, the temporal lag between changes in gene flow and accompanying changes in genetic differentiation may not be large (on the order of decades) for *L. decemlineata*. Temporal lags vary with migration rate, population size, generation time, and time since landscape change (Anderson et al., 2010; Epps & Keyghobadi, 2015; Landguth, Schwartz, McKelvey, & Luikart, 2010), and can persist for thousands of generations (Thomaz et al., 2017). Shorter temporal lags might be a common feature of agricultural insect pests and warrant further investigation in agricultural pest systems.

Demographic analysis suggested that our ability to detect landscape genetic associations was not complicated by the demographic histories of our study populations. Instead, any effects of potato land cover configuration might have been masked by the coarse resolution of our census data. An alternative argument might be that land cover had limited influence on gene flow among *L. decemlineata *populations. The negative correlations between genetic differentiation and landscape resistance in the Columbia Basin were highly influenced by one population that exhibited high genetic differentiation and low genetic diversity (“Tree” in Supporting Information Figures S2, S4–S7), suggesting our analysis distinguished very small landscape effects on genetic variation. However, estimates of landscape resistance effect sizes were robust to removal of this population, with BEDASSLE models actually recovering a stronger signal of contemporary landscape resistance on genetic differentiation when this population was excluded (Supporting Information Figure S4). Therefore, our results suggest there is evidence of genetic differentiation among *L. decemlineata *populations in association with contemporary versus historic potato land cover, though analyses using higher‐resolution data would be required to discern the importance of specific land cover types in shaping patterns of genetic variation.

### 4.2 Drivers of genetic differentiation and diversity

We found a significant, negative correlation between pairwise *F*_ST_ and nucleotide diversity among sites in the Central Sands and Columbia Basin, raising the question of whether this relationship is causal or driven by some external factor. Differences in nucleotide diversity could indicate that the strength of genetic drift varies among sites and that genetic differentiation is being driven by factors that reduce local effective population sizes. We found no evidence of an effect of the extent of potato land cover surrounding sites on genetic diversity, suggesting that the association is not driven by the amount of suitable habitat or the intensity of pest management. The severity and timing of founder events during colonization of agroecosystems could be an important determinant of genetic diversity, but we found no evidence of severe bottlenecks obviously associated with colonization history. Alternatively, these patterns of genetic diversity could be driven by ongoing landscape effects on gene flow, wherein populations isolated by dispersal‐restrictive land cover experience greater loss of nucleotide variation due to enhanced genetic drift (isolation by environment; Wang & Bradburd, 2014). Such a coupling of responses between landscape structure, genetic diversity, and differentiation has been observed in a specialist mammal (Balkenhol, Pardini, Cornelius, Fernandes, & Sommer, 2013), but not in two plant species (Pujol et al., 2017; da Silva Carvalho, Ribeiro, Côrtes, Galetti, & Collevatti, 2015). Numerous studies quantify associations between landscape structure and genetic diversity or population genetic differentiation, but rarely examine both jointly. Disentangling the effects of processes influencing gene flow and effective population size on measures of genetic differentiation and diversity represents an important challenge for future landscape genetic studies.

### 4.3 Historical landscape genetics of agricultural pests

An important caveat to inferring historic landscape effects from contemporary genetic samples is that geographic locations of populations are not stationary through time. The locations of agricultural pest populations shift each year due to crop rotation and insect dispersal, and might be very different from their locations in the mid‐1800s. Thus, estimates of historical landscape resistance between contemporary populations might only be moderately related to the ecological distance separating populations in the past. Though landscape genetic studies are beginning to use historic population samples to link changes in genetic structure with landscape change (Draheim, Moore, Fortin, & Scribner, 2018), this approach does not address the question of how historic landscape structure has shaped contemporary genetic structure. Future landscape genetic studies using county‐level historical land cover data could address the challenge of nonstationarity by extending the linear LRT metric to two dimensions, analogous to the least‐cost transect approach (Van Strien, Keller, & Holderegger, 2012).

The historical landscape genetic framework employed in this study is broadly applicable to other agricultural pests, including weeds and plant pathogens. Such studies might reveal general responses of pests to agricultural land‐use change or reveal life history traits that mediate the effects of past landscape structure on genetic differentiation and diversity. For example, genetic variation in more highly dispersive species (e.g., aphids) might be relatively insensitive to agricultural landscape configuration at most scales. Agricultural land use in the United States has changed substantially since the mid‐1800s and will likely continue to do so. Given an uncertain future, it will be important to learn how genetic variation in agricultural pests has been shaped by changing landscapes in the past in order to understand how it might be further shaped in the future.

## Data Availability

GBS reads are available in the Short Read Archive (accession no. SRP098822 and PRJNA508767), and potato land cover data are available from the Dryad Digital Repository (https://doi.org/10.5061/dryad.93481g3).
