# Supplementary material for: Patterns of genetic differentiation in Colorado potato beetle correlate with contemporary, not historic, potato land cover
Source: Evol Appl. 2019 Jan 12;12(4):804–14. doi: 10.1111/eva.12757 (PMC6439494; doi:10.1111/eva.12757)
Supplement: Supplementary file 1 [file EVA-12-804-s001.docx]

**Supplementary Material for:**

Changing agricultural landscapes drive genetic differentiation in Colorado potato beetle

**Table of Contents**

**Supplementary Tables**

**Table S1**. Locations, dates, and sample sizes of *L. decemlineata* populations collected between 2014-2015 from which genotyping-by-sequencing data was generated.

**Supplementary Figures**

**Fig. S1**. Genetic diversity (observed heterozygosity and nucleotide diversity) and average pairwise F_ST_ comparison between the Central Sands of Wisconsin and Columbia Basin of Oregon and Washington.

**Fig. S2**. Principle Components Analysis of allele frequencies (7,408 GBS-derived SNP loci) among *L. decemlineata* populations in (A) the Central Sands of Wisconsin and (B) the Columbia Basin of Oregon and Washington. Left: PC1 vs. PC2. Right: PC2 vs PC3.

**Fig. S3**. Change in potato land cover on a county-basis from 1850 to 2012 in the Central Sands (Wisconsin) and Columbia Basin (Oregon and Washington). Note that a common color scale was used for each region, masking small amounts of potato land cover in early censuses.

**Fig. S4**. (A) Relationship between landscape resistance (LRT) during the census periods of 1860, 1910, and 2012 and geographic distance among pairwise population comparisons for the Central Sands (top) and the Columbia Basin (bottom). (B) Relationship between landscape resistance estimates among census periods. Lines and statistical parameters represent the results of linear regression.

**Fig. S5**. Comparison of BEDASSLE estimates of effect size ratios (A) treating each census period independently (equivalent to Fig. 2b), (B) including all census periods in one model, and (C) treating each census period independently, but excluding the outlier Columbia Basin population ‘Tree’.

**Fig. S6**. Relationship between genetic differentiation (pairwise F_ST_) and geographic distance, and relationships between residual genetic differentiation (after accounting for effects of geographic distance) and landscape resistance in the census periods 1860, 1910, and 2012. Relationships are depicted including (A) and excluding (B) the Columbia Basin population ‘Tree’.

**Fig. S7**. Stairway plots depicting median (dark line) and 2.5^th^-97.5^th^ percentiles (light shading) of effective population size among 200 bootstrapped folded site frequency spectra. Inferences were based on the folded site (minor allele) frequency spectra for each *L. decemlineata* population, length of genomic coverage by genotyping-by-sequencing reads, an assumed mutation rate of 2.1x10^-9^ per site per generation, and a generation time of one year.

**Fig. S8**. Folded site (minor allele) frequency spectra for each *L. decemlineata* population. Columbia Basin populations are depicted in the left two columns, and Central Sands populations in the right.

**Supplementary Files**

**File S1**. R code used to create a PED file from SNP genotype data.

**Table S1**. Locations, dates, and sample sizes of *L. decemlineata* populations collected between 2014-2015 from which genotyping-by-sequencing data was generated.

| **Region** | **Site** | **Date** | **Latitude** | **Longitude** | **N** |  |
| --- | --- | --- | --- | --- | --- | --- |
| Columbia Basin | BOM | 5/17/2016 | 45.74437 | -119.61729 | 12 |  |
|  | CAR | 5/17/2016 | 45.97081 | -119.28996 | 12 |  |
|  | COL | 5/17/2016 | 45.79551 | -119.44032 | 12 |  |
|  | FOR | 5/16/2016 | 45.88300 | -119.40623 | 14 |  |
|  | HAR | 5/18/2016 | 45.81934 | -119.28216 | 12 |  |
|  | RDO | 6/17/2016 | 45.73573 | -119.84855 | 12 |  |
|  | TRE | 5/17/2016 | 45.78089 | -119.51342 | 10 |  |
|  | WAT | 5/18/2016 | 45.99272 | -119.74209 | 11 |  |
| Central Sands | BF4 | 6/9/2015 | 44.107967 | -89.795558 | 12 | |
|  | DkI | 6/4/2014 | 43.995781 | -89.600472 | 12 |  |
|  | H53 | 6/1/2015 | 44.081411 | -89.568900 | 10 |  |
|  | HM | 6/10/2015 | 44.651500 | -89.491058 | 11 |  |
|  | KB | 6/10/2015 | 44.545217 | -89.451383 | 11 |  |
|  | N62 | 6/5/2015 | 44.093497 | -90.052414 | 11 |  |
|  | P257 | 6/3/2015 | 44.167753 | -89.459608 | 12 |  |
|  | P745 | 6/12/2015 | 44.415931 | -89.553319 | 12 |  |
|  | Wys5 | 6/2/2015 | 44.225958 | -89.494386 | 10 |  |

**
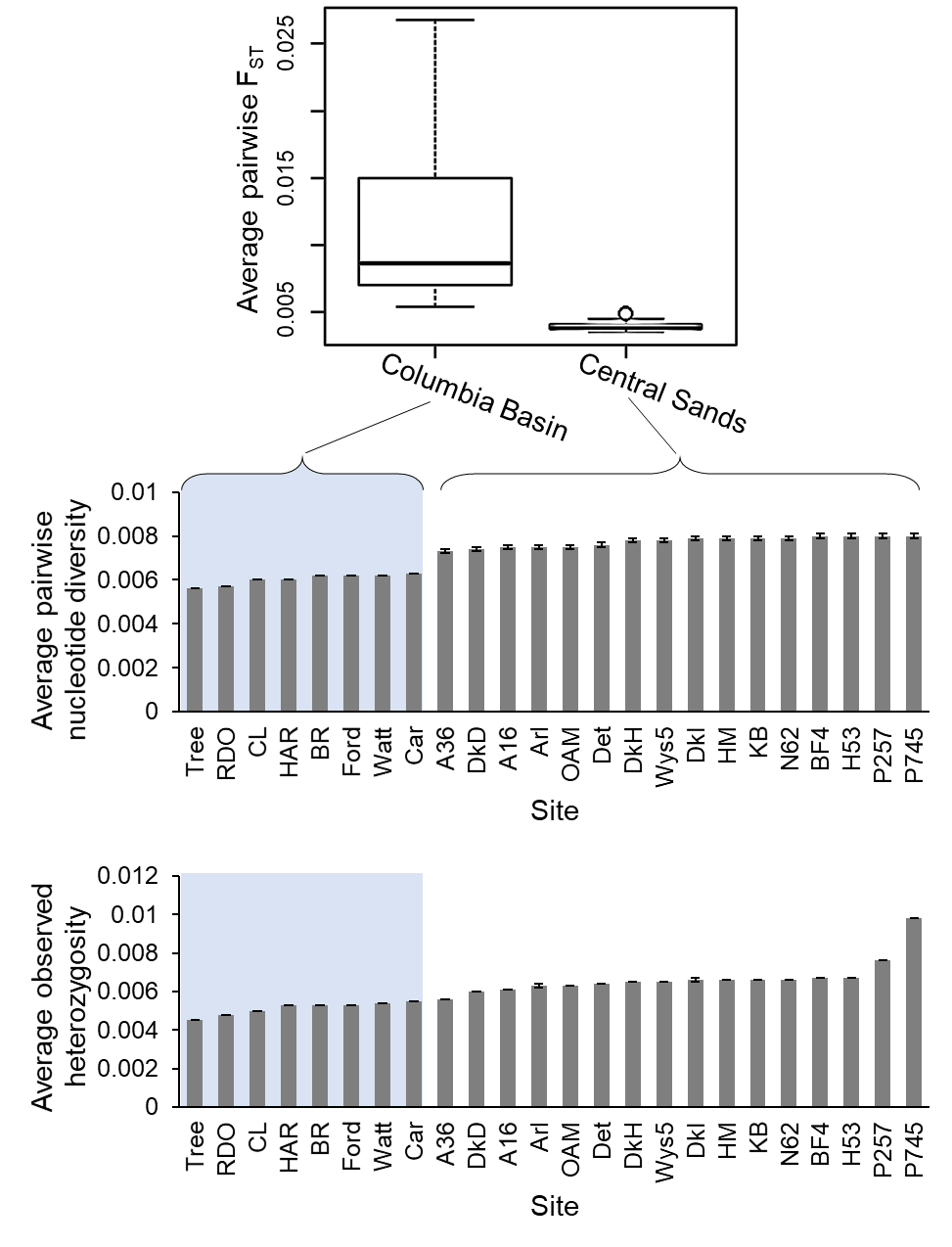
**

**Fig. S1**. Genetic diversity (average observed heterozygosity and average nucleotide diversity) and average pairwise F_ST_ comparison between the Central Sands of Wisconsin and Columbia Basin of Oregon and Washington.

**
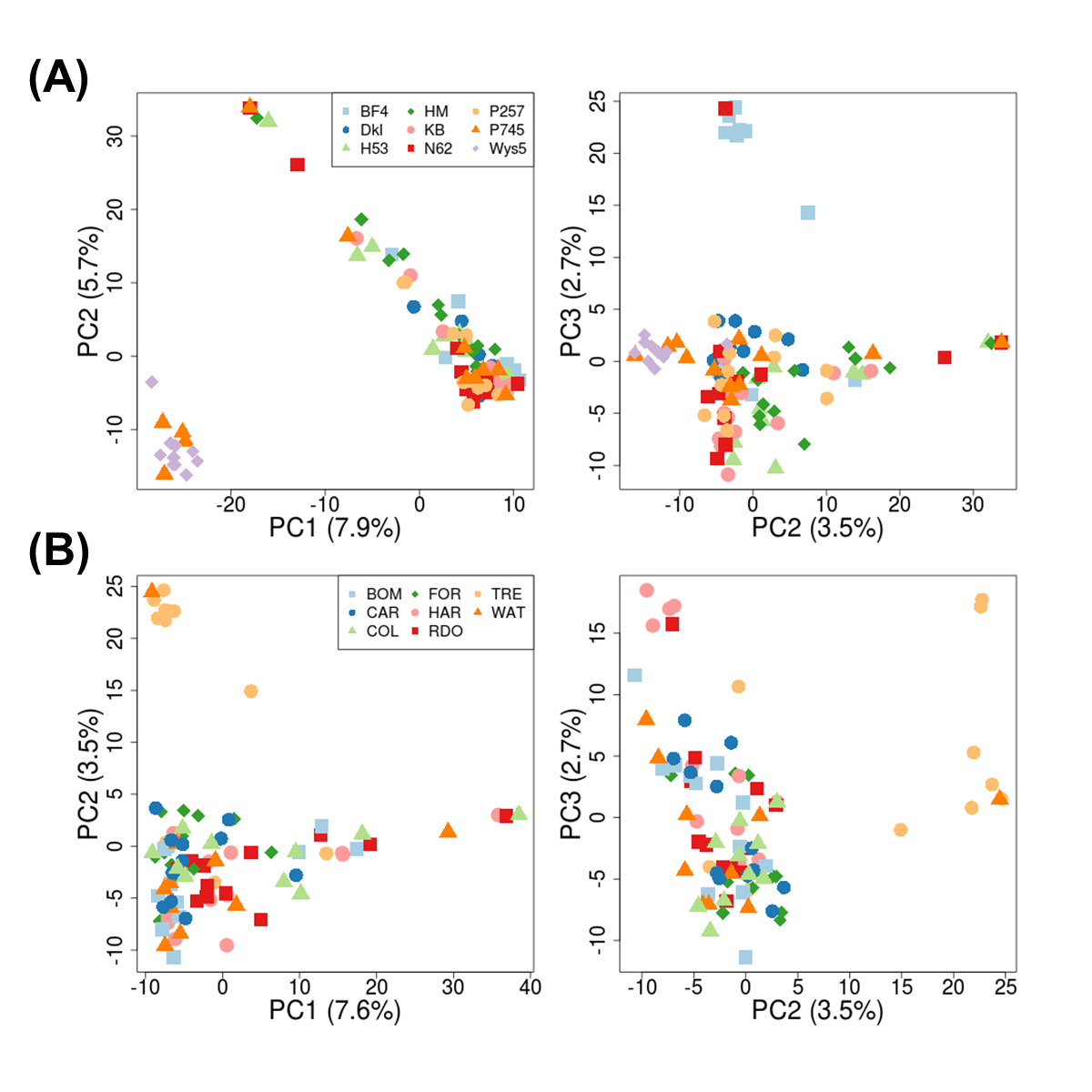
**

**Figure S2**. Principle Components Analysis of allele frequencies (7,408 GBS-derived SNP loci) among *L. decemlineata* populations in (A) the Central Sands of Wisconsin and (B) the Columbia Basin of Oregon and Washington. Left: PC1 vs. PC2. Right: PC2 vs PC3.


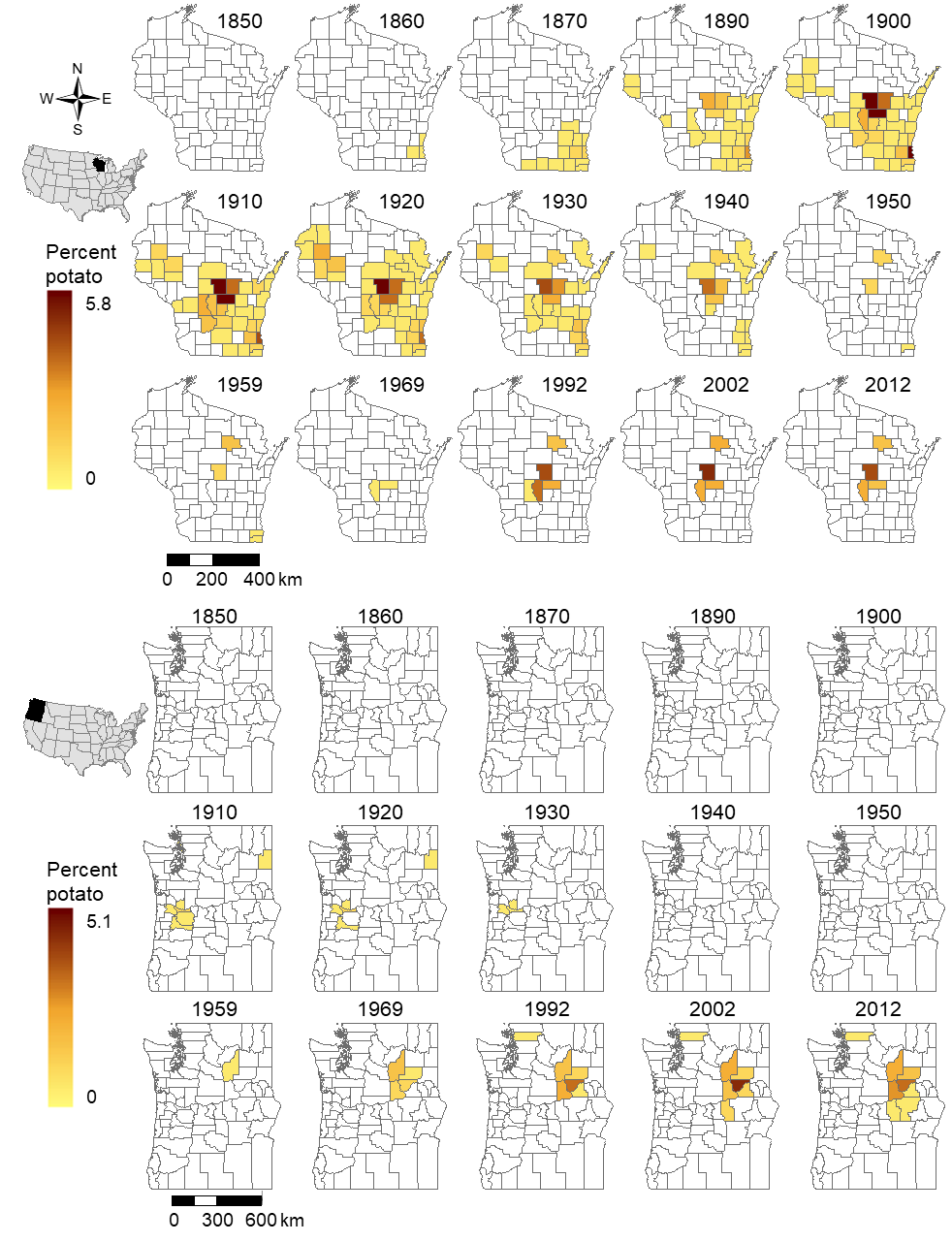


**Figure S3**. Change in potato land cover on a county-basis from 1850 to 2012 in the Central Sands (Wisconsin) and Columbia Basin (Oregon and Washington). Note that a common color scale was used for each region, masking small amounts of potato land cover in early censuses.

**
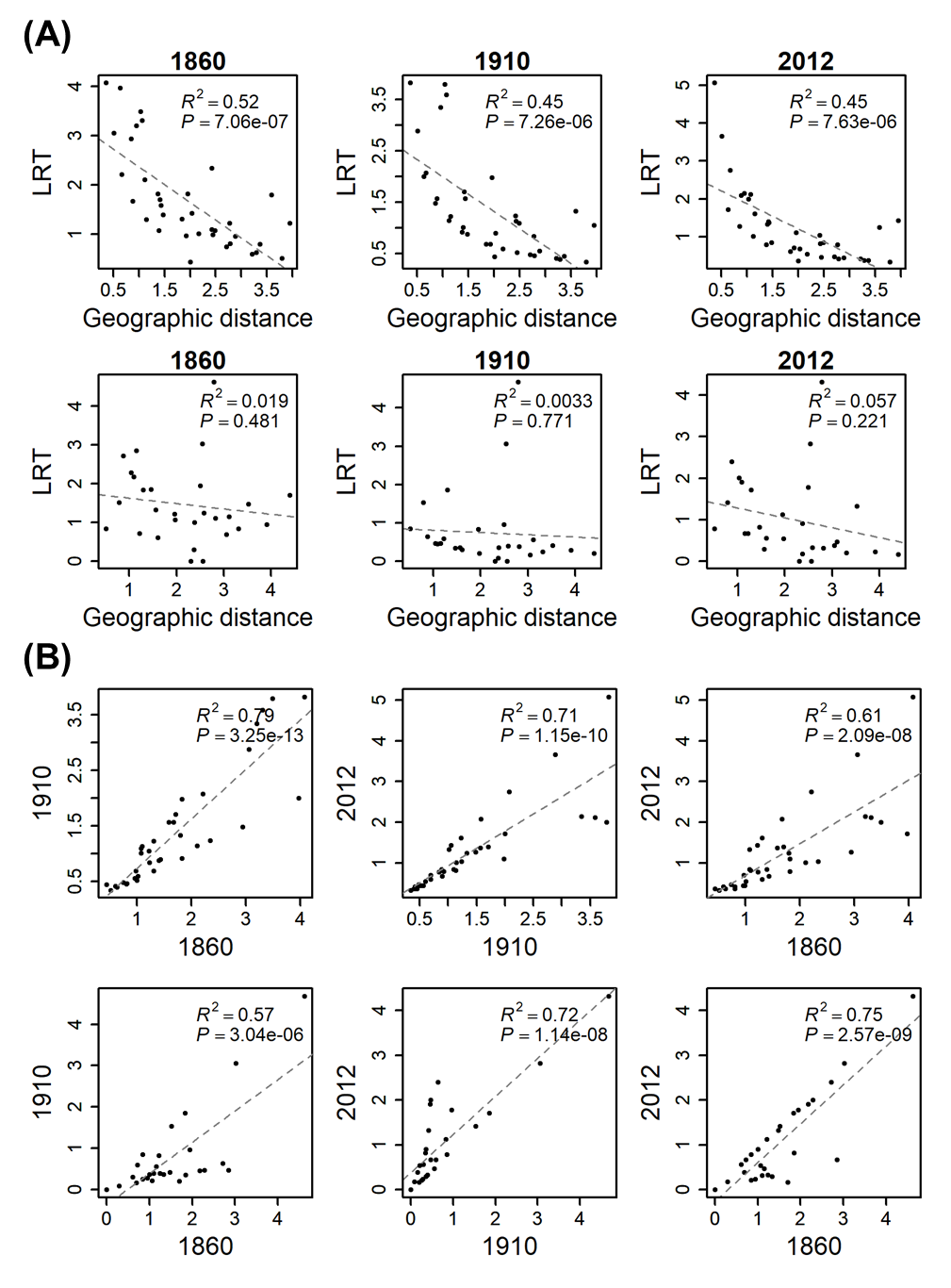
**

**Fig. S3**. (A) Relationship between landscape resistance (LRT) during the census periods of 1860, 1910, and 2012 and geographic distance among pairwise population comparisons for the Central Sands (top) and the Columbia Basin (bottom). (B) Relationship between landscape resistance estimates among census periods. Lines and statistical parameters represent the results of linear regression.


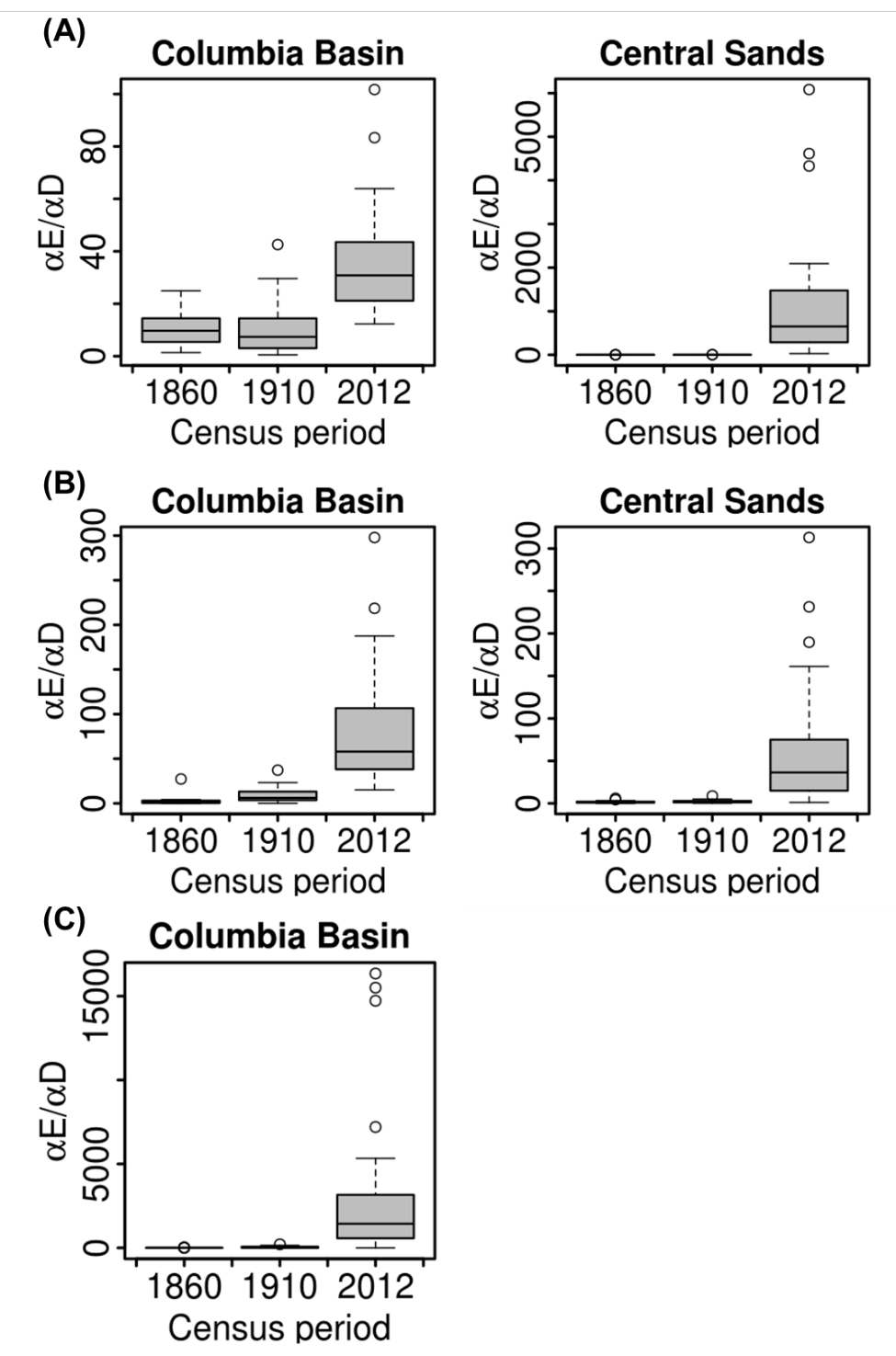


**Fig. S4**. Comparison of BEDASSLE estimates of effect size ratios (A) treating each census period independently (equivalent to Fig. 2b), (B) including all census periods in one model, and (C) treating each census period independently, but excluding the outlier Columbia Basin population ‘Tree’.


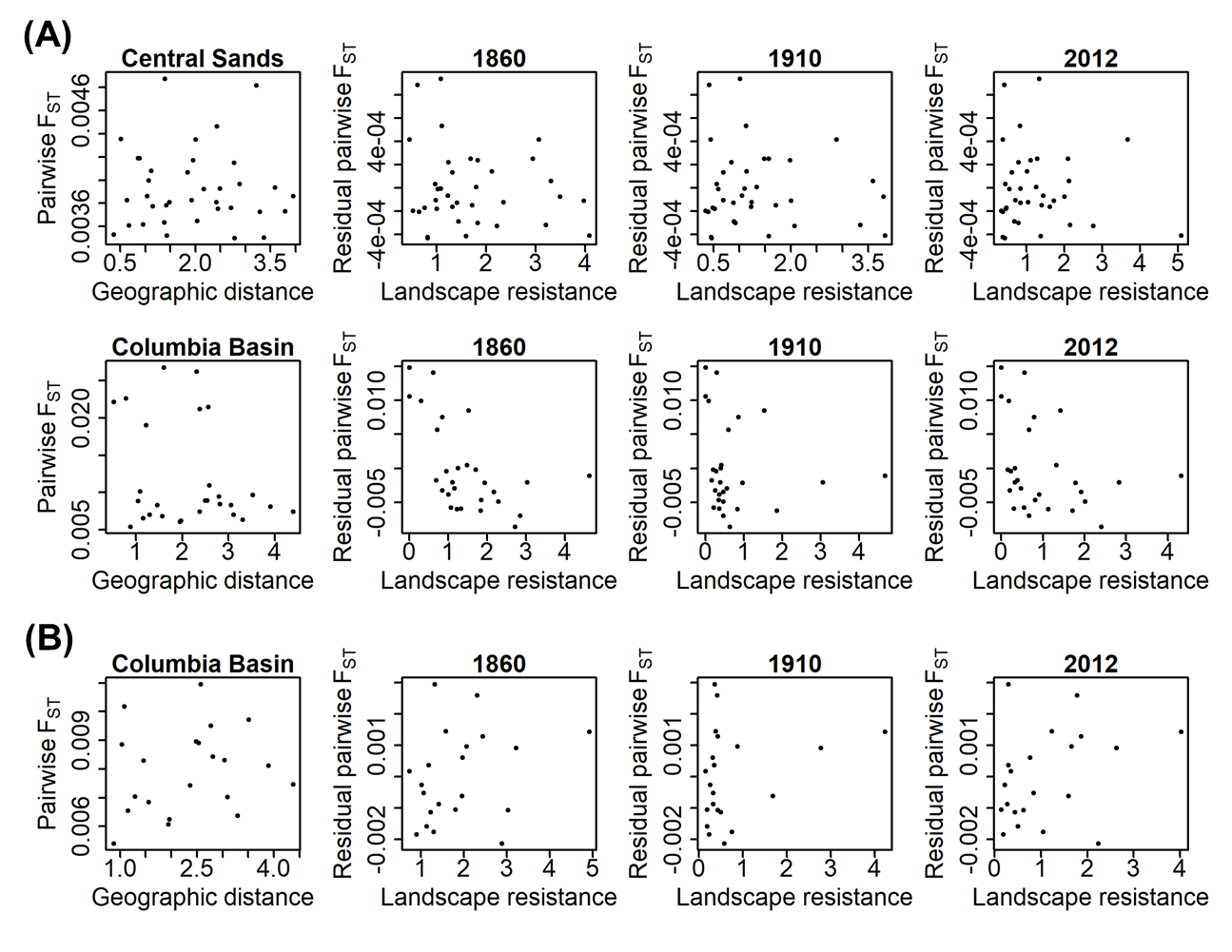


**Fig. S5**. Relationship between genetic differentiation (pairwise F_ST_) and geographic distance, and relationships between residual genetic differentiation (after accounting for effects of geographic distance) and landscape resistance in the census periods 1860, 1910, and 2012. Relationships are depicted including (A) and excluding (B) the Columbia Basin population ‘Tree’.


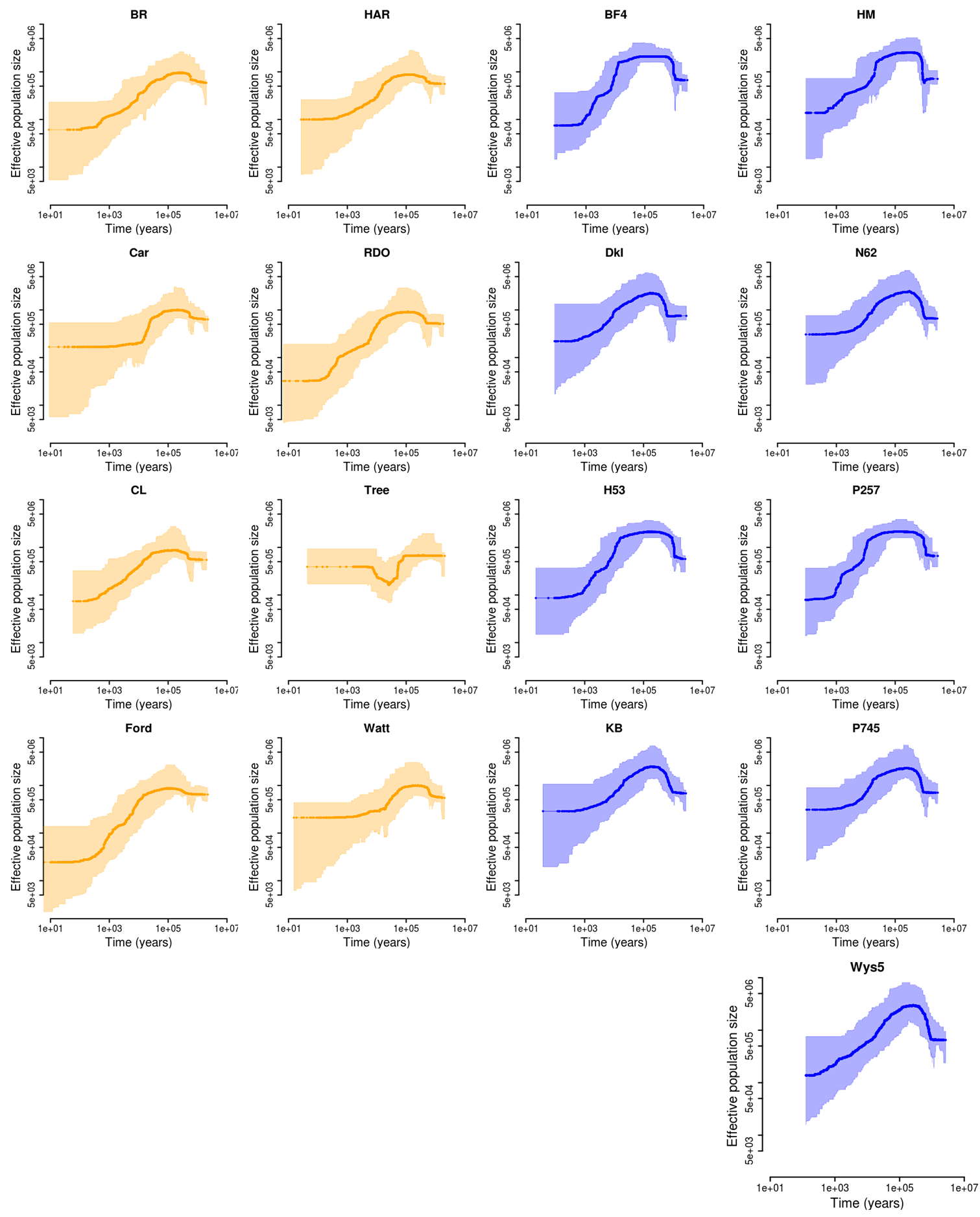


**Fig. S6**. Stairway plots depicting median (dark line) and 2.5^th^-97.5^th^ percentiles (light shading) of effective population size among 200 bootstrapped folded site frequency spectra. Inferences were based on the folded site (minor allele) frequency spectra for each *L. decemlineata* population, length of genomic coverage by genotyping-by-sequencing reads, an assumed mutation rate of 2.1x10^-9^ per site per generation, and a generation time of one year.

**
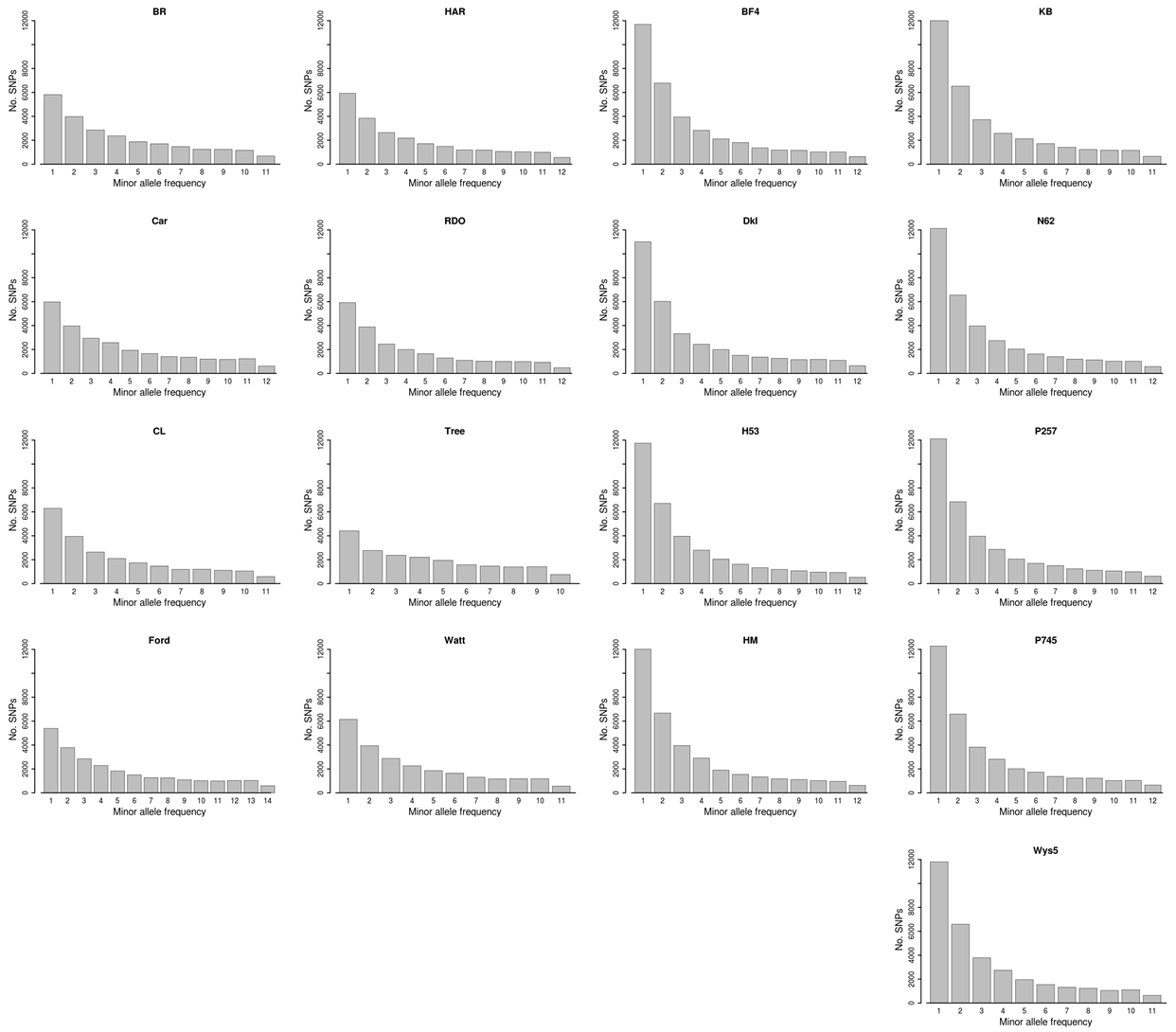
**

**Fig. S7**. Folded site (minor allele) frequency spectra for each *L. decemlineata* population. Columbia Basin populations are depicted in the left two columns, and Central Sands populations in the right.

**File S1**. R code used to create a PED file from SNP genotype data.

# Import genotype data

path.to.genotypes <- “” # file path (including file name) of a tab-delimited file containing a genotypes matrix with SNPs as rows and individuals as columns. Genotypes are encoded as homozygous reference = 0, heterozygous = 1, homozygous alternate = 2

path.to.pops <- “” # file path (including file name) to tab-delimited file with sample names in column 1 and population names in column 2

data <- read.table(path.to.genotypes,sep="\t")
metadata <- read.table(path.to.pops,sep="\t")

# Create PED file

# Define sample attributes (can leave blank for EEMS)
familyID <- matrix(metadata[,2][match(colnames(data),metadata[,1])])
individualID <- colnames(data)
paternalID <- rep(0,ncol(data))
maternalID <- rep(0,ncol(data))
sex <- rep(0,ncol(data))
phenotype <- rep(0,ncol(data))

ped <- matrix(NA,nrow=ncol(data),ncol=nrow(data)*2)
index <- seq(1,nrow(data)*2,2) #list of indeces for where to place alleles in the ped file
                             #counter to get index for pos list
for (i in 1:ncol(data)){ #for each individual
    k=1
    for (j in 1:nrow(data)){ #for each marker
        if (data[j,i]==0){ #account for each possible genotype, tell what number to place in each allele column
            ped[i,index[k]] <- 1      #first allele column
            ped[i,index[k]+1] <- 1    #second allele column
        } else if (data[j,i]==1){
            ped[i,index[k]] <- 1
            ped[i,index[k]+1] <- 2
        } else if (data[j,i]==2){
            ped[i,index[k]] <- 2
            ped[i,index[k]+1] <- 2
        } else {
            ped[i,index[k]] <- NA
            ped[riow,index[k]+1] <- NA
        }
        k=k+1
    }
}
PED <- cbind(familyID,individualID,paternalID,maternalID,sex,phenotype,ped)

path.to.ped <- “” # define location & file name to write PED output file (should end with “.ped”)
write.table(PED,path.to.ped,sep="\t",quote=F,row.names=F,col.names=F)

# Create MAP file
chrom <- matrix(NA,nrow=nrow(data),ncol=1)
for (i in 1:nrow(data)){
  chrom[i,1] <- strsplit(rownames(data)[i],"[.]",)[[1]][1]
}
SNPID <- rownames(data)
GD <- rep(0,nrow(data))
PD <- matrix(NA,nrow=nrow(data),ncol=1)

for (i in 1:nrow(data)){
    PD[i,1] <- strsplit(rownames(data)[i],"[.]",)[[1]][2]
}
MAP <- cbind(chrom,SNPID,GD,PD)
path.to.map <- “” # define location & file name to write MAP output file (should end with “.map”)

write.table(MAP,path.to.map,sep="\t",quote=F,row.names=F,col.names=F)
